# Action of Cinchona Alkaloids

**Published:** 1874-04

**Authors:** E. Buchanan Baxter

**Affiliations:** Medical Tutor in King’s College, London


*THE PRACTITIONER—London, November,* 1873 :

Action of Cinchona Alkaloids on Bacteria and Colorless Blood-Corpuscles.—*By E. Buchanan Baxter, M.D., Medical Tutor in King's College, London.*—Cinchona-bark and quinia are universally allowed to be antiperiodic, antiseptic and antiphlogistic. The first of these adjectives denotes their specific influence over the fevers and other morbid states caused by the paludal miasm; the second, their curative virtue in septic conditions of the blood; the third, their power to restrain the migration of the colorless blood-corpuscles from the vessels, and thereby to arrest, or at any rate to control the violence of suppurative inflammation.

These properties rest on evidence gathered at the bed-side. No sooner, however, do we quit the ground of clinical observation than our footing becomes insecure. What is the *nexus* between these therapeutic virtues? Do they depend on the same or on different properties of the alkaloid? There are two ways of arriving at a solution of this problem. One, by inquiring into the pathology of the diseases over which bark exerts a salutary power, and tracing some common factor in all of them which the drug may be supposed to influence. The other, by studying the action of the drug analytically, by investigating its effect on the simple processes of which the complex whole we term "disease" consists.

The singular freedom from decay enjoyed by all the varieties of cinchona bark must have been noticed by pharmaceutists from a very early period. The permanence of decoctions and infusions of bark, as compared *e.g.* with those of quassia, forces itself on the attention of the most careless dispenser. It was not till the middle of the last century, however, that the antiseptic qualities of bark were experimentally investigated. In the *Philosophical Transactions* for 1750, there is a paper by Sir John Pringle, entitled "Some Experiments on Substances resisting Putrefaction," giving a series of observations on the comparative antiseptic power of a variety of substances, including "the Bark." The author exposed bits of meat, steeped in solutions or decoctions of the antiseptics employed, to a temperature of 94°—104° F. for several days, and noted the kind and degree of putrefactive change which they underwent.

The experiment is described thus: "I made a strong decoction of the bark, and infused a piece of flesh in two ounces of it strained; which flesh never corrupted, though it remained two or three days in the furnace after the standard" (flesh steeped in brine) "was putrid."

Pringle's observations lay forgotten for upwards of a century. Even Briquet, in his elaborate and valuable monograph, refers very briefly to Pringle's results, and obviously confounds the antiseptic with the astringent properties of the bark.

No really adequate conclusions as to the antiseptic power of the cinchona alkaloids were possible so long as the intimate nature of the putrefactive process remained unknown. The circumstance that it depends on the evolution of a multitude of extremely simple organisms, narrows the area of the inquiry very materially. The question as to the antiseptic properties of quinia and other substances resolves itself into the question of their action upon microzymes. * * * *

Experiments show that all the cinchona alkaloids possess antiseptic virtues, and that the quantitative difference between them is not very marked. Arranging them in the order of their efficacy, quinia and quinidine stand in the first rank; cinchonidine next; and last, cinchonia.

It is interesting to note that of the four alkaloids, cinchonia is the only one which is not fluorescent. Is this physico-chemical property correlated in any way with antiseptic power? The following experiment with æsculin, the active principle of the horse-chestnut bark, shows that it is not; or, at any rate, that a substance more fluorescent than quinia possesses no antiseptic virtue comparable with that of the cinchona alkaloids. * * *

These experiments seem to show that the cinchona alkaloids, in greater proportion than is needful to arrest the multiplication of microzymes, exert no appreciable influence on the growth of *Penicillium.* That the same proportion of mercuric chloride renders the spores absolutely barren. That sodic sulphite and hyposulphite are without any effect; but that the latter, in acid solutions, yielding free sulphur and sulphur dioxide, appears to check the development of the spores.

Quinia, as an antiseptic, has thus been compared with—

1. Mercuric chloride, sodic sulphocarbolate and strychnia—substances to which antiseptic properties are ascribed.

2. Berberin, beberia, picrate of potassium, quinidine, cinchonia, cinchonidine, æsculin—substances credited with antiperiodic virtues.

3. Sodic Sulphite and hyposulphite—to which both antiseptic and antiperiodic properties are attributed.

The following thoughts are suggested by the results of this comparison:

1. Quinia is doubtless excelled by other antiseptics, but there is no substance equal to it in antiseptic power which can be introduced into the blood in the same proportions without risk of fatal effects, if we except the other cinchona alkaloids and the sulphate of beberia.

2. Quinia, in such fractional doses as are capable of being introduced into the circulation, exerts an inhibitory, not a toxic, action upon microzymes. It *may* check septic changes; it cannot destroy the organisms to which such changes are due.

3. The four cinchona alkaloids are very nearly equal in antiseptic power. Arranged in the strict order of their efficacy, they stand thus: Quinia=quinidine; next comes cinchonidine; last, though at no great distance, cinchonia.

4. It is a singular circumstance that this order corresponds to that in which the four alkaloids are arranged in the Report of the Madras Cinchona Commission in 1868, with reference, not to their antiseptic, but to their *antiperiodic* power, as determined by clinical experience. This report establishes:

(*a.*) That quinidine equals quinia in febrifuge action.

(*b.*) That cinchonidine is only slightly less efficacious.

(*c.*) That cinchonia, though somewhat inferior, is, notwithstanding, a valuable remedial agent in fever.

This agreement may possibly be due to coincidence; if not, it seems to afford some countenance to the zymotic theory of the paludal miasm; or it would perhaps be more correct to say that it does not *oppose* it. It really shows no more than that the one property is in some way correlated with the other; it does not prove their identity.

5. Among reputed antiperiodics, the sulphate of beberia seems to equal quinia in antiseptic power.

6. Among reputed antiseptics, sodic sulphocarbolate and strychnia have a decided value, though they stand some way below quinia.

7. Sodic sulphite has a feeble, though decided, antiseptic power; sodic hyposulphite little or none.

8. Berberin and æsculin are hardly, if at all, antiseptic.

9. Potassic picrate is a strong antiseptic, almost, if not quite, equal to quinia. It is doubtful, however, whether it can be administered in sufficient doses without danger to life.

10. The action of inhibitory drugs on the vitality of microzymes affords no clue to their action on *Penicillium.* Substances like mercuric chloride, which are immediately fatal to the former, arrest the growth of the latter when employed in the same minute proportions.

The toxic action of quinia on the colorless corpuscles of the blood was first noticed by Professor Binz; and his observations have since been extended and corrobarated by other investigators. This effect of alkaloids acquires a special significance in view of recent doctrines of inflammation and suppuration; the emigration of leucocytes from the vessels into the inflamed tissue giving rise to the whole, or a varying fraction of the total inflammatory exudation. Any substance capable of arresting or retarding the movements of the corpuscles might therefore be expected to arrest or retard the local consequences of tissue-irritation; and it is probable that the proliferative activity of the autochthonous corpuscular elements, depending as it does on properties common to them with the white corpuscles of the blood, may be restrained by the same means that are found effectual in checking the migratory propensities of the latter. It seemed desirable to ascertain if the other cinchona alkaloids were endowed with similar propties in an equal degree.

Experiments seem to show that the four cinchona alkaloids are able speedily to arrest the *migratory* movement of the colorless corpuscles of newt's blood, in the proportion of 1 in 1,500. That the quantitative differences between them are not well marked, quinia appearing to stand first in the order of power. That the proportion of the alkaloid necessary to arrest the movement of the waxy protuberances is much larger than that which arrests migration and the putting forth of filamentous processes. And this would lead us to expect that it may be possible to check the migratory propensities of the colorless corpuscles while in the body, without impairing, or permanently abolishing, their vitality—that they may be narcotized, so to speak, without being killed.

Sulphate of beberia, which approaches the cinchona alkaloids so closely in antiseptic power, is thus seen to rival them in its inhibitory action upon the *migratory* movement of the colorless corpuscles. The effect of its solutions in defining the nuclei of the red discs—an effect not produced by the cinchona alkaloids—leads one to suspect that the mode of action is somewhat different in the two cases. Strychnia has some influence upon the colorless corpuscles; but it stands very far behind beberia and the cinchona alkaloids.

Experiments seem to show that potassic picrate, standing almost on a level with the cinchona alkaloids in antiseptic power, exerts a comparatively feeble influence over the spontaneous movements of the colorless corpuscles. It cannot, therefore, be asserted, at present, that the antiseptic and the antiphlogistic actions of a drug are necessarily connected with each other.

Experiments with æsculin prove that it exerts no very marked effect on the migratory activity of the blood corpuscles.

